# Adaptive Navigation Algorithm with Deep Learning for Autonomous Underwater Vehicle

**DOI:** 10.3390/s21196406

**Published:** 2021-09-25

**Authors:** Hui Ma, Xiaokai Mu, Bo He

**Affiliations:** 1 Shanghai Marine Electronic Equipment Research Institute, Shanghai 201108, China; qinmahui@163.com; 2 College of Shipbuilding Engineering, Harbin Engineering University, Harbin 150001, China; 3 Qingdao Innovation and Development Center, Harbin Engineering University, Qingdao 266000, China; 4 College of Information Science and Engineering, Ocean University of China, Qingdao 266000, China; bhe@ouc.edu.cn

**Keywords:** autonomous underwater vehicle, navigation algorithm, deep learning, variational Bayesian

## Abstract

Precise navigation is essential for autonomous underwater vehicles (AUVs). The measurement deviation of the navigation sensors, especially the microelectromechanical systems (MEMS) sensors, is a crucial factor that affects the localization accuracy. Deep learning is a novel method to solve this problem. However, the calculation cycle and robustness of the deep learning method may be insufficient in practical application. This paper proposes an adaptive navigation algorithm with deep learning to address these questions and realize accurate navigation. Firstly, this algorithm uses deep learning to generate low-frequency position information to correct the error accumulation of the navigation system. Secondly, the χ2 rule is selected to judge if the Doppler velocity log (DVL) measurement fails, which could avoid interference from DVL outliers. Thirdly, the adaptive filter, based on the variational Bayesian (VB) method, is employed to estimate the navigation information simultaneous with the measurement covariance, improving navigation accuracy even more. The experimental results, based on AUV field data, show that the proposed algorithm could realize robust navigation performance and significantly improve position accuracy.

## 1. Introduction

The autonomous underwater vehicle (AUV) is an essential equipment for ocean exploration and has been widely applied in recent years. By collecting information from various relevant sensors, the navigation system could estimate the position, velocity, and other navigation information of the AUV [1,2]. Therefore, AUV navigation technology is an essential prerequisite for ocean exploration. Because the global positioning system (GPS) is invalid when the AUV operates underwater, it is necessary to develop underwater autonomous navigation technology. The main navigation methods at present include inertial navigation [3,4], acoustic navigation [5,6], simultaneous localization and mapping (SLAM) [7,8], geophysical map-based navigation [9], and integrated navigation [10]. Among the above methods, integrated navigation is the most widely used navigation method for AUVs.

Considering the cost and convenience, the integrated navigation system for the small-scale AUV, which works in shallow seas and lakes, mainly relays on the Doppler velocity log (DVL) and the attitude and heading reference system (AHRS). Based on the kinematics equation, the dead-reckoning method can be used to calculate the location of AUVs [11,12]. However, the measurement error, which could be considered Gaussian white noise, would cause the position error accumulation. The navigation system needs to use the filter to reduce the interference of measurement error. The regularly used filters include extended the Kalman filter (EKF) [13,14], and the unscented Kalman filter (UKF) [15,16]. EKF is the most commonly used nonlinear filtering method for AUV navigation. By intercepting the first-order Taylor series, it could approximate the nonlinear transformation process, but the EKF neglects the higher-order terms, which leads to the system model error. The Gauss-Newton is an optimized method that could reduce the nonlinear model error to a certain extent [17]. Optimized methods, such as genetic algorithm [18] and particle filter [19], could also be used to improve the performance of EKF. UKF uses an unscented transformation to sample and estimate the state by calculating the covariance of the system state and the observed state, which could achieve a higher estimation accuracy than EKF. However, in practical application, the computational complexity of UKF is high, and it is easy to introduce the problem of nonpositive covariance, which also affects the robustness of the navigation system.

To achieve the state estimation, accurate error covariance is necessary. Because of the influence of environmental factors and sensor characteristics, the measurement noise covariance is uncertain, even time-varying, and it is difficult to obtain precise noise errors in engineering applications. The existing solution assumes the noise satisfies the white Gaussian noise distribution and realizes the state estimation through an adaptive filter. Sage Husa [20,21], H∞ filter [22], the maximum likelihood estimation method [23,24], and variational Bayesian (VB) [25,26] are the primary adaptive algorithms at present. The adaptive filter could obtain the state estimation with approximate error covariance.

The adaptive filter could effectively estimate the error distribution in the case of white noise. However, due to the magnetic sensitivity of the external ferromagnetic material and the electromagnetic wave of AUVs, the data of AHRS may have a deviation from the true value, which could be treated as a color noise. The color noise could not be addressed by the conventional method and it is still a challenging issue in practical applications. Deep learning [27] has developed rapidly in recent years and has been applied in image processing [28], language translation [29], pattern recognition [30], and other engineering fields [31]. Since the neural network method could approach the optimal solution, this method could effectively improve the navigation accuracy when the sensor has a large deviation. However, according to our research, the positioning accuracy of the neural network method is unsatisfactory compared to that of the traditional navigation method when the sensor accuracy is high [32]. Additionally, the navigation information frequency of the deep learning method is low since the calculation cycle of the deep learning method needs data from a duration time, which would cause this method to be insufficient in some missions.

This paper proposes an adaptive navigation algorithm with deep learning, which could be employed to the integration of AHRS and DVL. Firstly, the method employs the VB method to adapt the covariance of the measurement of noise covariance. Subsequently, when the calculation cycle of deep learning is finished, the neural network would obtain a relatively precise position, and the measurement model of the filter uses this position, and the data from AHRS and DVL, to correct the system state. Otherwise, the measurement value includes the data of AHRS and DVL. During the impact from the environment and AUV motion, the DVL measurement may fail [33,34]. To avoid the impact of DVL outliers on the filter estimate, the χ2 rule is employed to evaluate the DVL measurement [35,36]. When the DVL measurement fails, the observation will remove the data from the DVL. The proposed method is evaluated by the AUV field data, and experimental results show that the algorithm proposed in this paper could significantly improve the accuracy and the robustness of the navigation system.

The remainder of this paper is organized as follows: Section 2 introduces the navigation system of the platform and the conventional EKF navigation method. The details of the proposed method are presented in Section 3. Section 4 presents the performance of different navigation methods and the experimental results analysis. Finally, the conclusion of this work is in Section 5.

## 2. Navigation System

### 2.1. Equipment and Sensors

**Figure 1:**
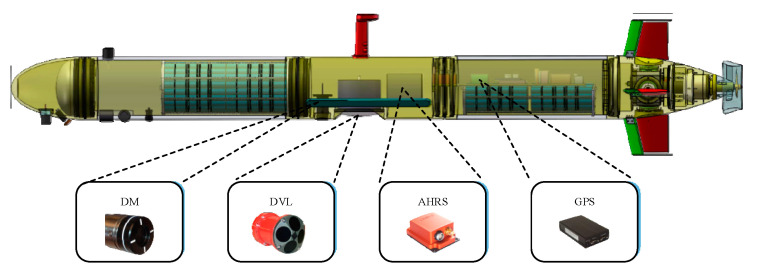
Structure of the Sailfish autonomous underwater vehicle (AUV).

Figure 1 is the structure of the Sailfish AUV. The Sailfish AUV includes the propulsion system, communication system, environmental perception system, and navigation system. The propulsion system includes the propeller, rudder, and their related control units, which provide the power of AUV motion. The communication system aims to provide stable information interaction between the AUV and the offshore control unit. The environmental perception system uses sensors to obtain environmental information that can be used for mission performance and obstacle avoidance. Among them, the navigation system could provide navigation information for other systems, so it is the fundamental function of AUVs. To realize the navigation state estimation, the AUV is equipped with various sensors and equipment. The navigation system is integrated in the middle cable of the whole AUV. The related sensors and equipment include a GPS, DVL, AHRS, and a depth meter (DM). Each sensor and equipment will be introduced in the following parts. The specification of part sensors and equipment could refer to our previous work [37].

As a piece of passive positioning equipment, the GPS could obtain an accurate position when the AUV floats on the surface. The GPS calculates its localization by receiving the signal of the satellite, and the position information has no error accumulation. However, because of the interference from the wind and waves, the GPS navigation information may have outliers. A filter is needed before the GPS information can used in the navigation system. Table 1 presents the specifications of the GPS.

The DVL is aimed at measuring the linear velocity of the AUV. When the DVL works, a short sound pulse with a fixed frequency will be transmitted and reflected by the bottom of the seafloor. The transducer of the DVL calculates the linear velocity based on the frequency change of the echo. The sound echo would be interference with environment and the AUV motion state, which caused the DVL measurement to fail in practical application. When the DVL operates normally, the measurement error is considered to be the white noise. Table 2 presents the specifications of the DVL.

The AHRS, which includes a three-axial gyroscope, accelerometer, and magnetic compass, could directly measure the linear acceleration, angular velocity, and magnetic intensity. The above data would be a fusion with a Kalman filter to get a stable heading and attitude. However, due to the magnetic sensitivity of the external ferromagnetic material and the electromagnetic wave of AUV, the information of AHRS, especially the yaw data, may have a deviation from the true value, which could not be treated as white noise. Table 3 presents the specifications of AHRS.

The DM is an essential sensor that can provide AUV depth during vehicle operation in the water. The core component of the DM is a pressure sensor that can obtain the intensity of pressure. The micro process unit of the DM uses the pressure, fluid density, and constant of gravitation to calculate the depth of the AUV. The data from DMs are relatively accurate and can be employed directly in AUV navigation. Therefore, the main challenge in our AUV navigation is to achieve a position estimation in the horizontal coordinate. Table 4 presents the specifications of the DM.

### 2.2. Conventional EKF Navigation Algorithm

The conventional navigation algorithm of the AUV platform is EKF. EKF can be divided into two parts: time update and measurement update. In the time update stage, the system employs the kinematics equation to predict the system state, and the Jacobin matrix of the system update function is used to forecast the covariance of system noise. Equations (1) and (2) represent the system propagate process:

(1) $$X_{k|k-1}=f\left(X_{k-1}\right)$$

(2) $$P_{k|k-1}=F_{k}P_{k-1}F_{k}^{T}+Q$$

where *X* is the system state, *f* is the system update function based on the kinematics equation, *P* is the system error covariance, *Q* is the covariance of the system noise, and *F* is the system noise transition matrix.

In the measurement update stage, the system state and covariance would be corrected by the measurement value. The Kalman gain, which is determined by the covariance of system noise and measurement noise, represents the degree of correction. The measurement update process is shown in the following equations:

(3) $$K_{k}=P_{k|k-1}H_{k}^{T}{\left(H_{k}P_{k|k-1}H_{k}^{T}+R_{k}\right)}^{-1}$$

(4) $$X_{k}=X_{k|k-1}+K_{k}(Z_{k}-h(X_{k|k-1}))$$

(5) $$P_{k}=(I-K_{k}H_{k})P_{k|k-1}$$

where *K* is the Kalman gain, *Z* is the measurement vector, *h* is the measurement function, *H* is the Jacobin matrix of measurement function, and *R* is the measurement noise covariance matrix.

In the AUV navigation modeling process, the kinematics equation of AUV navigation is represented as Equation (6):

(6) $${\left[\begin{array}{c} x\\ y\\ \psi \\ u\\ v\\ a_{u}\\ a_{v}\\ \omega \end{array}\right]}_{k}={\left[\begin{array}{c} x+(ut+0.5a_{u}t^{2})\cos(\psi )-(vt+0.5a_{v}t^{2})\sin(\psi )+n_{x}\\ y+(ut+0.5a_{u}t^{2})\sin(\psi )+(vt+0.5a_{v}t^{2})\cos(\psi )+n_{y}\\ \psi +\omega t+n_{\psi }\\ u+a_{u}t+n_{u}\\ v+a_{v}t+n_{v}\\ a_{u}+n_{au}\\ a_{v}+n_{av}\\ \omega +n_{\omega } \end{array}\right]}_{k-1}$$

where *x* and *y* are the positions along the north and east axis, *Ψ* is the heading of AUV, *u* and *v* are the longitudinal and transverse velocities in horizontal coordinates, *a_u_* and *a_v_* are the related acceleration, *ω* is the angular velocity of heading, and *t* is the time interval. Since the error propagation is a complex coupled process, to simplify the calculations, the system noise is assumed as the white Gaussian noise. The system noise is shown as Equation (7).

(7) $$n={\left[\begin{array}{cccccccc} n_{x} & n_{y} & n_{\psi } & n_{u} & n_{v} & n_{au} & n_{av} & n_{w} \end{array}\right]}^{T}$$

As the system update function is nonlinear, the system noise transition function, which is shown in Equation (8), is the Jacobin matrix of the system update function.

(8) $$F=\left[\begin{array}{cccccccc} 1 & 0 & -(ut+0.5a_{u}t^{2})\sin(\psi )-(vt+0.5a_{v}t^{2})\cos(\psi ) & t\cos(\psi ) & -t\sin(\psi ) & 0.5t^{2}\cos(\psi ) & -0.5t^{2}\sin(\psi ) & 0\\ 0 & 1 & \left(ut+0.5a_{u}t^{2})\cos(\psi )-(vt+0.5a_{v}t^{2})\sin(\psi \right) & t\sin(\psi ) & t\cos(\psi ) & 0.5t^{2}\sin(\psi ) & 0.5t^{2}\cos(\psi ) & 0\\ 0 & 0 & 1 & 0 & 0 & 0 & 0 & t\\ 0 & 0 & 0 & 1 & 0 & t & 0 & 0\\ 0 & 0 & 0 & 0 & 1 & 0 & t & 0\\ 0 & 0 & 0 & 0 & 0 & 1 & 0 & 0\\ 0 & 0 & 0 & 0 & 0 & 0 & 1 & 0\\ 0 & 0 & 0 & 0 & 0 & 0 & 0 & 1 \end{array}\right]$$

The measurement state includes the velocity, acceleration, heading, and angular velocity and is shown in Equation (9):

(9) $$Z={\left[\begin{array}{cccccc} \psi & u & v & a_{u} & a_{v} & w \end{array}\right]}^{T}$$

As the measurement state has a linear relationship to the system state, the measurement transition matrix is easy to express:

(10) $$H=\left[\begin{array}{cc} 0_{6\times 2} & I_{6\times 6} \end{array}\right]$$

According to the above navigation system modeling, the navigation information of the AUV can be calculated in real-time by using the EKF algorithm. However, the data of the AHRS is vulnerable to the external ferromagnetic material and the electromagnetic waves of the AUV. In the actual AUV application, the noise of the heading is colored noise and difficult to estimate, which is the main reason that affects the positioning accuracy of the navigation system. The velocity measurement error of the DVL is another factor that affects navigation accuracy.

## 3. Adaptive Navigation Algorithm with Deep Learning

**Figure 2:**
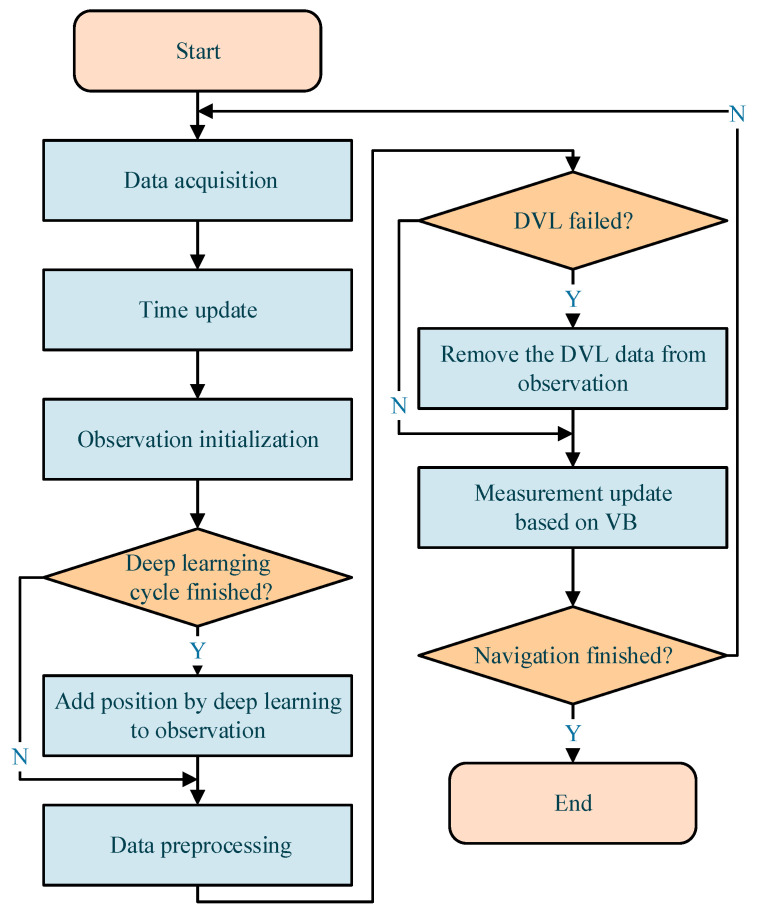
Flowchart of the developed algorithm.

This paper proposed an adaptive navigation algorithm with deep learning to address sensor noise in the navigation system. Figure 2 is the flowchart of the developed method. The algorithm is based on the EKF method, and regular observation contains the data of the DVL and AHRS. When the deep learning calculation cycle is finished, the position calculated by deep learning would be added to the observation *Z_D_*. To avoid interference from DVL outliers, the observation *Z_A_* would remove the data of DVL when it fails. The VB method is employed in the EKF to adjust the covariance of noise.

### 3.1. Deep Learning Navigation Method

**Figure 3:**
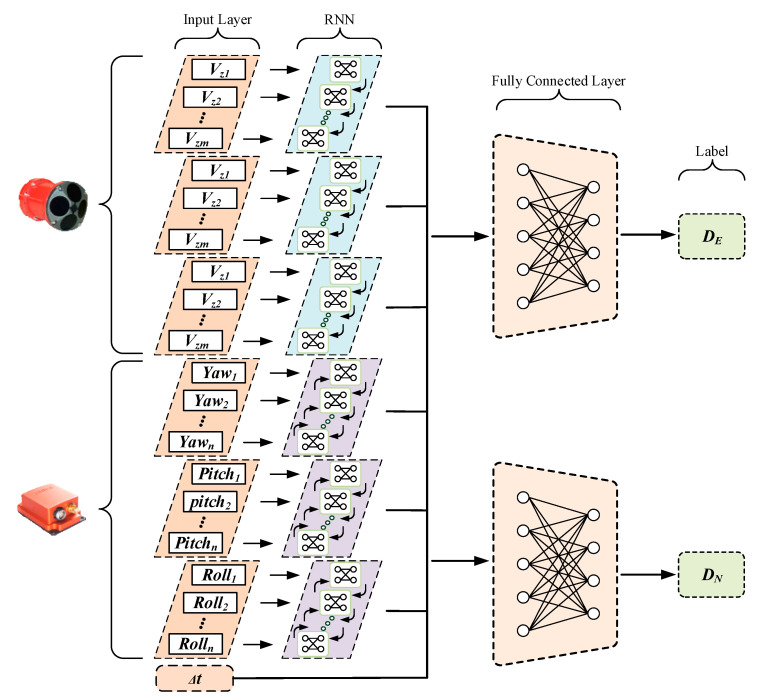
Structure of hybrid recurrent neural networks (RNNs).

**Figure 4:**
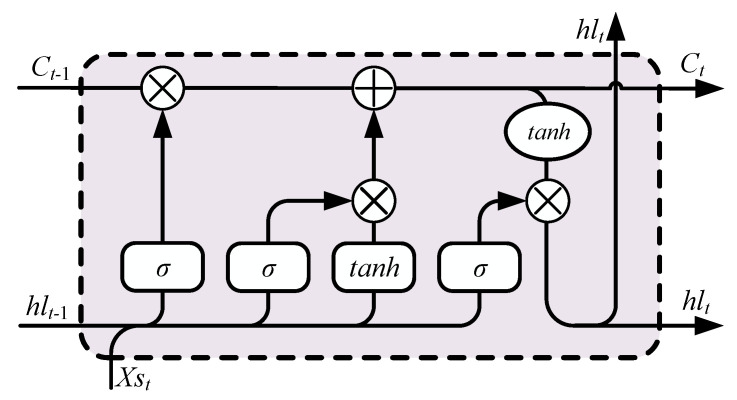
Structure of long short-term memory (LSTM).

The yaw data of AHRS includes colored noise, which is hard to estimate by the conventional method. Therefore, we employed a hybrid recurrent neural networks (RNNs) framework to realize AUV position estimation. Since the training process uses the GPS movement as the label, and the raw data of the sensors as the input, the trained framework could include the interference from yaw error [32]. Figure 3 presents the structure of hybrid RNNs. This framework uses unidirectional and bidirectional long short-term memory (LSTM) to handle different sensor data. The calculation process of LSTM is as follows [38]. Figure 4 is the structure of LSTM.

(11) $$fg_{t}=\sigma_{f}\left(W_{f}\left[hl_{t-1},Xs_{t}\right]+b_{f}\right)$$

(12) $$ig_{t}=\sigma_{i}\left(W_{i}\left[hl_{t-1},Xs_{t}\right]+b_{i}\right)$$

(13) $$C_{t}=fg_{t}\circ C_{t-1}+ig_{t}\circ \tanh\left(W_{c}\left[hl_{t-1},Xs_{t}\right]+b_{c}\right)$$

(14) $$og_{t}=\sigma_{o}\left(W_{o}\left[hl_{t-1},Xs_{t}\right]+b_{o}\right)$$

(15) $$hl_{t}=og_{t}\circ \tanh\left(C_{t}\right)$$

where *Xs* is the sequence input of data, the *W* and *b* are the weight and bias, the *fg* is the forgetting gate, *ig* is the input gate, *C* is the cell state, *Og* is the output gate, and *hl* is the hidden layer. The activation function *σ* uses the sigmoid. The ∘ represents the point-wise product operation.

The RNN structure is used to preprocess the raw data of the DVL and AHRS. Then the output of different RNNs and the time interval are used as the input to transform the displacement by a fully connected layer [39]. The GPS trajectory is smoothed by an adaptive fault-tolerance filter and separated into segments to generate labels for training. In this network, root mean square error (RMSE) is selected to calculate the loss between the label and prediction. The RMSE calculation process is shown in Equation (16).

(16) $$\xi =\sqrt{\frac{1}{m}\sum_{i=1}^{m}{\left(\chi_{i}-Xd_{i}\right)}^{2}}$$

where *m* is the number of train datasets, *χ* is the label, and *Xd* is the predicted value of deep learning.

To improve the training efficiency of the network, the activation function in the fully connected layers employs the rectified linear unit (ReLu) to overcome the vanishing gradient problem. According to our experiments, the learning rate should be reduced during the training process, so the adaptive gradient (AdaGrad) is selected to adjust the learning rate, and AdaGard could effectively decrease training cycles. The trained network could achieve AUV position estimation.

Deep learning is an end-to-end navigation method and does not need to handle various complex matrix operations. Therefore, it is easy to implement. Since the neural network method could approximate the optimal solution, it could obtain relatively precise localization when the raw data has a large deviation. However, the performance is relatively weak when the raw data is accurate. The calculation cycle of the deep learning method needs data from a duration time, which causes the deep learning method to only obtain navigation information with a low frequency. In this work, the position information of deep learning is used as the element in the observation to correct the navigation information. Here the measurement vector *Zd* includes the deep learning output and the data from the DVL and AHRS. The RMSE of deep learning describes the deviation degree from truth value, and the square of RMSE is mean square error (MSE), which is the same as the meaning of measurement variance. Hence, we use the MSE as an approximate measurement variance of the neural network position estimation. The measurement noise covariance matrix *Rd* concludes the MSE of deep learning and the measurement noise. Because the variance obtained by the MSE is still not accurate, it needs to be corrected by the VB method.

### 3.2. DVL Fault Diagnosis

When AUVs cruise in the water, the motion state is interfered with by the waves and surge, which may have an adverse impact on the DVL measurement. The moving objects near the DVL, or a rapid change in the terrain, is another factor for DVL accuracy.

To restrain the above impact, the χ^2^ rule is selected to judge if the DVL measurement fails [35,36]. The innovation of DVL measurement is satisfied with white noise when the data of DVL is available.

(17) $$r_{DVL}\sim (0,N_{DVL})$$

In our navigation system, the covariance matrix *N_DVL_* is expressed as follows:

(18) $$N_{DVL}={\left[HP_{k|k-1}H^{T}+R\right]}_{2:3,2:3}$$

The fault detection criterion is defined as Equation (19).

(19) $$C_{DVL}=r_{DVL}{}^{T}{\left(N_{DVL}\right)}^{-1}r_{DVL}$$

If $C_{DVL}$ is above the threshold, we consider the DVL measurement to have failed, and the DVL measurement is removed from the observation matrix *Za*. The measurement noise covariance matrix *Ra* only contains the measurement noise of AHRS. The χ2 rule is suitable for mutant fault detection, which could effectively avoid the impact of DVL measurement failure in navigation.

### 3.3. Variational Bayesian Method

The accuracy of the covariance estimation is significant to the performance of the Kalman filter. Since the system covariance is the inherent characteristics, and approximate estimation could maintain the stability of the filter, the measurement covariance is the main factor that affects the accuracy of the state estimation. In our navigation algorithm, the measurement includes position, yaw angle, and velocity. Among them, the covariance of position is obtained by deep learning, which is an approximate value, and the covariance of the DVL would be affected by the environment. The VB method is introduced to the state estimation to simultaneously estimate the measurement covariance to address this question.

The VB method aims to obtain the conditional probability density of joint distribution *p*(*X_k_*, *R_k_*|*Z_k_*). To simplify the calculation process, the joint distribution is approximate to the product of two independent probability densities, as shown in Equation (20).

(20) $$p(X_{k},R_{k}|Z_{k})\approx q(X_{k})q(R_{k})$$

According to the VB theory, the approximate probability density can be obtained by minimizing the Kullback-Leibler divergence from the actual probability density. Since the measurement follows the law of normal distribution, the covariance of covariance is assumed to satisfy the inverse Wishart distribution [37]. The VB method can be concluded as follows.

In the time update process, the VB method is similar to the standard Kalman filter. In addition, the parameter initialization is as Equation (21).

(21) $$\left\{ \begin{array}{l} \alpha_{k|k-1}=\rho (\alpha_{k-1}-m-1)+m+1\\ \beta_{k|k-1}=\rho \beta_{k-1} \end{array}\right.$$

where *α* and *β* are the elements in the probabilistic distribution of measurement covariance, *m* is the degree of the observation matrix, and *ρ* is the factor that approximates the posterior of the measurement covariance.

The measurement update is an iterative process. The calculation is expressed in Equation (22).

(22) $$\left\{ \begin{array}{l} \alpha_{k}^{\left(i+1\right)}=\alpha_{k}^{\left(i\right)}+1\\ \beta_{k}^{\left(i+1\right)}=\beta_{k}^{\left(i\right)}+{\left(Z_{k}-HX_{k}^{\left(i\right)}\right)}^{T}(Z_{k}-HX_{k}^{\left(i\right)})+HP_{k|k-1}H^{T}\\ R_{k}^{\left(i+1\right)}={\left[(\alpha^{\left(i+1\right)}-m-1)/\beta^{\left(i+1\right)}\right]}^{-1}\\ K^{\left(i+1\right)}=P_{k|k-1}H^{T}{\left(HP_{k|k-1}H^{T}+R_{k}^{\left(i+1\right)}\right)}^{-1}\\ X_{k}^{\left(i+1\right)}=X_{k|k-1}+K^{\left(i+1\right)}(Z_{k}-HX_{k|k-1})\\ P_{k}^{\left(i+1\right)}=P_{k|k-1}-K^{\left(i+1\right)}HP_{k|k-1} \end{array}\right.$$

where *i* represents the number of iterations. After the iteration is finished, the last system state and measurement covariance are the estimations of the VB method.

In the proposed navigation algorithm, the covariance of measurement is uncertain, which could seriously impact navigation accuracy. The VB method could simultaneously estimate the measurement noise covariance and system state, which could reduce the interference from imprecise covariance.

For simplicity, the notation of the AUV model and the developed algorithm is regrouped for clarity in Table 5.

## 4. Experiments and Analysis

**Figure 5:**
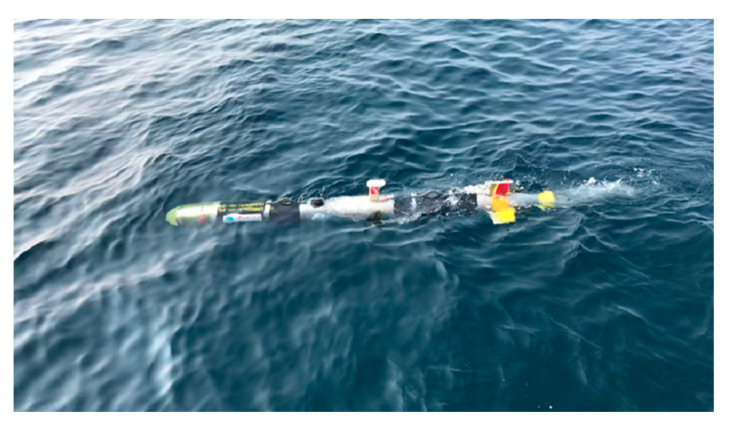
Experiment scene of AUV platform.

In this section, a series of experiments based on the AUV field data is carried out. To evaluate the performance of different navigation methods, a truth value, such as GPS position, is necessary for the experiment platform. As the AUV could not obtain a GPS position while immersed underwater, the field data were collected when the AUV was cruising on the surface. Moreover, the GPS-smoothed trajectories could be generated as the labels to train the deep learning network. Figure 5 is the Sailfish AUV during the Tuandao Bay experiments. The scene is the Sailfish cruising on the surface.

The field data of our experiments were acquired from different places, such as the Menlou Reservoir, Jiaozhou Bay, and Tuandao Bay. Table 6 depicts the details of the experimental data. The field data of four groups covers straight lines, turns, and cycles, which represent almost all of the motion modes of AUVs. Additionally, the experimental environment in different places is varied. The winds and waves in bay and coastal waters are more intense than those in the reservoir.

**Figure 6:**
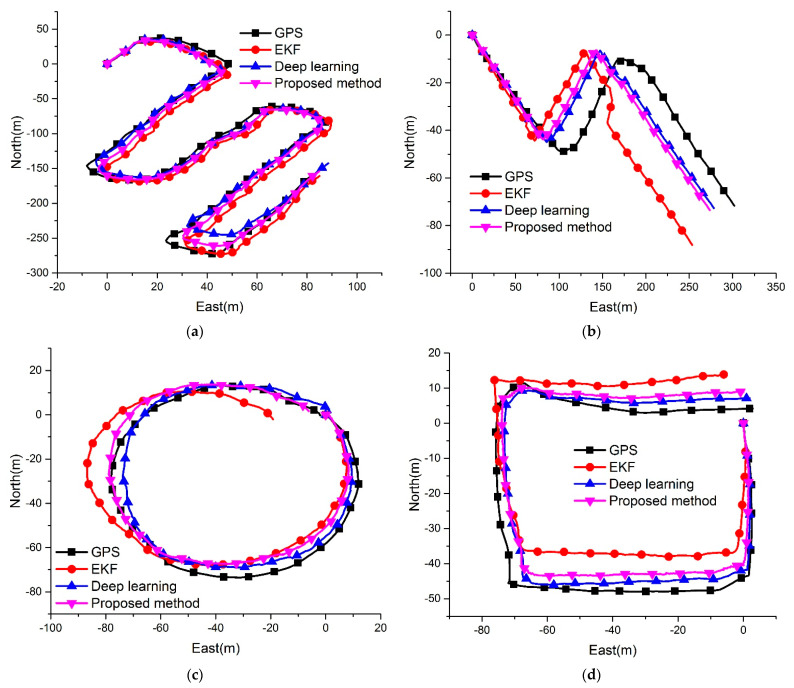
Four experimental results of the ground truth trajectory and the trajectories obtained using different methods based on field data.

In our experiments, the conventional EKF, pure deep learning, and the proposed algorithm were employed to generate the AUV trajectory. Figure 6 shows the paths produced by various algorithms. Since the GPS data frequency in our platform is 1 Hz, the GPS trajectory is divided into movements per second. Therefore, the navigation data frequency of deep learning is 1 Hz, while the conventional method and the proposed method are 10 Hz.

In Figure 6, black lines represent the smoothed GPS trajectories that are considered as the ground truth, red lines represent the conventional EKF trajectories, blue lines represent pure deep learning trajectories, and violet lines represent the proposed method. According to our previous research, the performance of the deep learning method would be better than the conventional EKF algorithm in most cases. However, the EKF trajectory would be closer to the ground truth when the accuracy of navigation sensors is high, which caused the pure deep learning in Test1 to be worse than the EKF. The proposed method could adaptively fuse the data from sensors and the deep learning method by the VB method. Therefore, the navigation accuracy in Test1 can be improved more than other methods. In Test2, DVL raw data has a jump that causes the EKF to deviate from the expected trajectory. The deep learning method is robust to the outliers and could effectively avoid interference. In the proposed method, the DVL fault diagnosis method detects the measurement fails and removes the velocity data from the observation, so the proposed method could maintain the robustness towards the measurement outliers. Test3 and Test4 show that the proposed method could effectively improve navigation accuracy more than the conventional EKF method.

**Figure 7:**
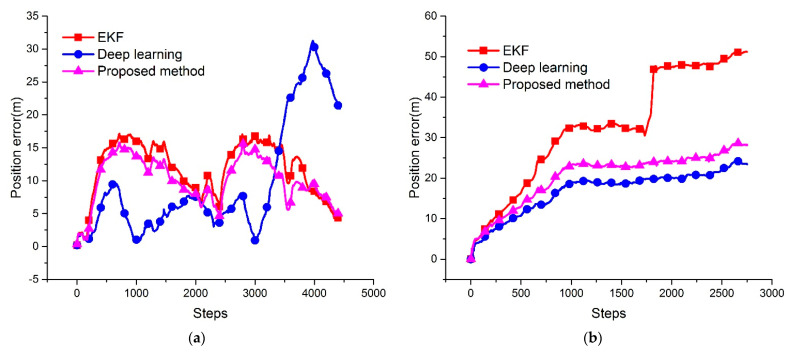

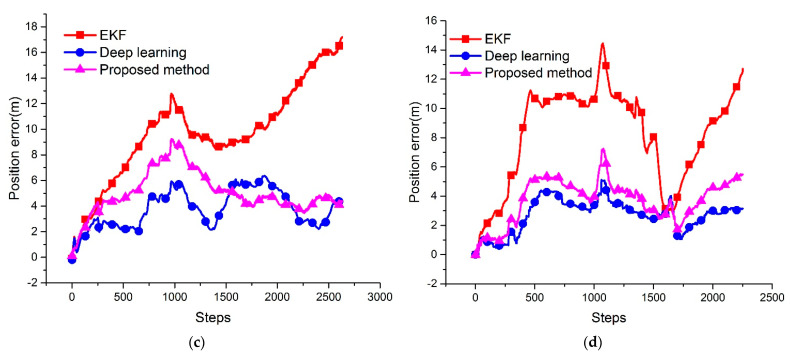
Position error between the ground truth and the estimation of different navigation methods.

Figure 7 shows the position errors between the ground truth and different algorithms. The position errors are the distance between the GPS and the estimation results of different methods. In Figure 7a, because of the slight sensor deviation and the data fusion strategy, the performance of the proposed method is better than other methods. Figure 7b shows the error of Test2. Since the DVL measurement has outliers, the deep learning method could significantly improve the navigation accuracy. The proposed method, with a fault diagnosis function leading to the algorithm, has positive fault tolerance ability to the DVL measurement fails. Figure 7c,d show that the proposed method could improve the position accuracy compared to the conventional EKF method, and the accuracy is close to the deep learning method. Table 7 summarizes the RMSE of all the above algorithms during the four experiments. The RMSE results evidence that, although the proposed algorithm is insufficient compared to deep learning in most cases, it could achieve norm frequency and robust navigation, and improve accuracy to a larger extent than the conventional method. A number of experimental tests verify that the RMSE could improve by at least 14.4%.

## 5. Conclusions

In this work, we developed an adaptive navigation algorithm based on deep learning. Firstly, this algorithm uses deep learning to generate low-frequency position information to correct the navigation error. Secondly, the χ2 rule is selected to judge if the DVL measurement fails, which could avoid the interference from DVL outliers. Thirdly, the adaptive filter based on the VB method is employed to estimate navigation information simultaneous to the measurement covariance, improving navigation accuracy even more.

Different from the pure deep learning navigation method, this work could achieve robustness and high accuracy navigation with a normal frequency, which could be satisfied by the requirements of various missions. The experimental results based on AUV field data verified that even the performance of the proposed algorithm is slightly worse than pure deep learning. However, it has good robustness and could effectively improve navigation accuracy compared to the conventional navigation algorithms. In the future, we will carry on more complex integrated navigation system design, such as the integration of different acoustic equipment, and investigate the performance of the proposed algorithm.

## Figures and Tables

**Table 1:** Specification of GPS used on the AUV platform.

Equipment Type	GPS
Position Accuracy	3 m (CEP50)
Velocity Accuracy	0.05 m/s
Update Rate	5 Hz (Max)

**Table 2:** Specification of DVL used on the AUV platform.

Scheme 600	DVL
Sound frequency	600 KHz
Velocity accuracy	1%
Operational height	0.3–110 m

**Table 3:** Specification of AHRS used on the AUV platform.

Sensor Type	AHRS
Yaw accuracy	1°
Pitch/Roll accuracy	0.3°
Output frequency	Up to 2 kHz
Bias stability (Gyroscopes)	10°/h
Bias stability (Accelerometor)	15 μg

**Table 4:** Specification of DM used on the AUV platform.

Sensor Type	DM
Resolution	0.001% range
Accuracy	0.01% range
Update Rate	5 Hz
Response Time	1 millisecond

**Table 5:** AUV model and developed algorithm notation.

Variable	Description
*X*	State vector
*f*	State predict function
*F*	State predict transition matrix (Jacohian matrix of *f*)
*Z*	Measurement vector (data of DVL and AHRS)
*h*	Measurement function
*H*	Measurement transition matrix (Jacohian matrix of *h*)
*n*	White Gaussian noise.
*R*	Measurement noise covariance matrix
*K*	Kalman gain
*Xs*	Sequence data input of RNN
*W*, *b*	Weight and bias of neural cell
*fg*	Forgetting gate
*ig*	Input gate
*Og*	Output gate
*hl*	Hidden layer
*σ*	Activation function (sigmoid function usually)
*Zd*	Measurement vector includes deep learning data
*Rd*	Measurement noise covariance matrix includes deep learning
*Za*	Measurement vector of AHRS
*Ra*	Measurement noise covariance matrix of AHRS

**Table 6:** Details of AUV field data used in experiments.

No.	Start Point	End Point	Distance
Test1	120.34096° E, 36.16952° N	120.34219° E, 36.16920° N	751 m
Test2	121.20101° E, 37.40701° N	121.20509° E, 36.40629° N	316 m
Test3	120.29453° E, 36.05187° N	120.29454° E, 36.05187° N	260 m
Test4	120.29362° E, 36.05155° N	120.29351° E, 36.05159° N	224 m

**Table 7:** Root mean square error (RMSE) of position in different navigation methods.

No.	EKF	Deep Learning	Proposed Method
Test1	10.6899	13.0804	9.3048
Test2	33.6729	10.8476	13.1595
Test3	7.2525	4.0369	5.3061
Test4	7.6301	2.9252	4.0130
